# On-site detection system of *Candidatus* Liberibacter asiaticus by using TaqMan probe-based insulated isothermal polymerase chain reaction (iiPCR)

**DOI:** 10.1371/journal.pone.0287699

**Published:** 2023-06-23

**Authors:** Shun-Min Yao, Meng-Ling Wu, Ting-Hsuan Hung

**Affiliations:** 1 Department of Plant Pathology and Microbiology, National Taiwan University, Taipei, Taiwan; 2 Division of Forest Protection, Taiwan Forestry Research Institute, Taipei, Taiwan; 3 Research Center for Plant Medicine, National Taiwan University, Taipei, Taiwan; University of Saskatchewan College of Agriculture and Bioresources, CANADA

## Abstract

Citrus Huanglongbing (HLB) is one of the most destructive diseases of citrus plants caused by the obligate and phloem-limiting bacterium *Candidatus* Liberibacter asiaticus (Las). Reliable detection methods are important for successful control of the disease. This study was aimed to develop a rapid and user-friendly on-site detection system for Las using the TaqMan probe-based insulated isothermal polymerase chain reaction (iiPCR) assay. The Las-specific on-site detection system could be completed within one hour by simple DNA extraction coupled with a portable POCKIT device, which can perform PCR amplification and automatically provide qualitative results derived from fluorescence signals. The sensitivity of the TaqMan probe-iiPCR assay could be as low as single copy of Las, comparable to a real-time PCR method. Further testing of the field citrus samples showed 100% agreement between the TaqMan probe-iiPCR assay and the real-time PCR method, and the on-site detection system also demonstrated a great performance of Las detection. With high specificity and sensitivity, the on-site detection system developed in this study becomes a simple, rapid and powerful tool for detecting Las in fields.

## Introduction

Citrus Huanglongbing (HLB), also known as citrus greening, is one of the most destructive diseases of citrus worldwide and it affects most of commercial citrus cultivars [1, 2]. HLB has caused serious damage to the citrus industry by reducing the fruit quality and yield as well as shortening tree lifespan [2, 3]. HLB-affected trees commonly express symptoms including irregular yellow mottling, hardening, vein yellowing, and vein corking of leaves and incomplete coloring of mature fruit [1, 4, 5]. The causal agents of HLB were identified as Gram-negative α-proteobacteria: ‘*Candidatus* Liberibacter asiaticus’ (Las) from Asia, ‘*Ca*. L. africanus’ (Laf) from Africa and ‘*Ca*. L. americanus’ (Lam) from South America [6–8]. HLB can be spread by vegetative propagation and insect vectors. For the latter, the Asian citrus psyllid (ACP), *Diaphorina citri*, is the main vector in Asia and America, whereas the African psyllid, *Trioza erytreae*, is the main vector in Africa [9–11]. Several diagnosis techniques for HLB such as bioassays, enzyme-linked immunosorbent assay (ELISA), polymerase chain reaction (PCR), real-time PCR and loop-mediated isothermal amplification (LAMP) have been developed [12–15]. Due to the low concentration and uneven distribution of Las in citrus plant [16], PCR-based methods are more commonly used for detection. However, conventional PCR and the requirement of gel electrophoresis for detection of PCR products are relatively time-consuming.

Insulated isothermal PCR (iiPCR), which is established on the basis of Rayleigh-Benard convection, can be carried out in the specially designed R-tube^TM^ (GeneReach, Taiwan) in an insulated isothermal device. The iiPCR assay has been shown to be rapid, low-cost and sensitive for pathogens detection [17–19]. Temperature gradients can be generated and stably maintained in the R-tube; therefore, the three thermocycle steps (denaturation, annealing and extension) simultaneously take place by circulating the ingredients through the temperature gradients [18, 19]. Recently, the POCKIT Nucleic Acid Analyzer (GeneReach, Taiwan) and the hand-held POCKIT Micro Plus Nucleic Acid Analyzer (GeneReach) are designed for performing iiPCR assay, and automatically detecting and interpreting results by sensing fluorescent probe signals within one hour [20]. This POCKIT system has been demonstrated as a useful tool for sensitive and specific detection of several important pathogens in animals, humans and plants, such as equine influenza virus H3N8, severe acute respiratory syndrome coronavirus 2 (SARS-CoV-2), Zika virus and *Fusarium oxysporum* f. sp. *cubense* race 4 [21–24].

In this study, a time-saving HLB diagnostic protocol based on TaqMan-iiPCR assay was developed for the on-site detection system of Las in HLB-infected plants. Using this sensitive Las-detection method, HLB could be accurately monitored by routine screenings to facilitate control of HLB.

## Materials and methods

### Plant samples preparation

All experimental citrus plants were kept in insect-free greenhouses. The citrus plants of major cultivars in Taiwan including Ponkan mandarin (PM, *Citrus reticulata*), Liucheng sweet orange (LSO, *C*. *sinensis*), Eureka lemon (EL, *C*. *limon*) and Wentan pummelo (WP, *C*. *grandis*) confirmed as free from Las by PCR were used as healthy control. For HLB inoculation, healthy citrus plants were grafted by Las-infected scions. The inoculated plants with HLB foliar symptoms were confirmed by PCR detection, and these inoculated plants were included as the positive control [25].

### Conventional DNA extraction

The DNA was extracted from plant tissues with slightly modified method according to Hung et al. [25]. Leaf midrib (0.5 g) were ground thoroughly in liquid nitrogen. The powder was mixed with 2.7 mL extraction buffer (0.1 M Tris-HCl, 0.1 M EDTA, 0.25 M NaCl, pH8.0) and 0.3 mL 10% sarkosyl (N-Lauroylsarcosine) for 1 h incubation at 55°C. After 6,000 *g* centrifugation (5 min), 800 μL supernatant was collected. 100 μL of 5 M NaCl and 100 μL CTAB (10% CTAB in 0.7 M NaCl) were added and the mixture was incubated at 65°C for 10 min. The mixture was treated with 600 μL chloroform/isoamyl alcohol (24:1), centrifuged at 12,000 *g* for 5 min. The supernatant (850 μL) was collected and treated with 600 μL phenol/chloroform/isoamyl alcohol (25:24:1). After 12,000 *g* centrifugation (5 min), 600 μL supernatant was collected and mixed with 360 μL isopropanol for precipitating the nucleic acid. The pellets collected after centrifugation at 12,000 *g* for 10 min were washed with 70% ethanol, dried, and resuspended in 100 μL TE buffer (pH 8.0).

### Simple DNA extraction

The part of a leaf midrib (approximately 4 cm, same as the length of an Eppendorf tube) was cut into 30 pieces and mixed with 200 μL Extraction buffer (0.1 M Tris-HCl, 0.1 M EDTA, 0.25 M NaCl, pH8.0), 50 μL 0.5N NaOH and 50 μL 1% SDS. After incubation for 3 min, the sap was diluted with TE buffer to serve as the template for iiPCR.

To optimize the simple DNA extraction method, different dilution ratios (5-folds, 10-folds, 20-folds and 50-folds) were evaluated by a conventional PCR as following: 25 μL PCR reaction mixture containing 2 μL template DNA, 1X PCR buffer, 0.2 mM dNTPs, 400 nM plant 18S rRNA-encoding gene primer pair [26] and 1 unit of Super Therm Taq DNA polymerase (BERTEC, Taiwan). Thermolcycle condition included: 95°C for 5 min; 30 cycles at 95°C for 30 s, 60°C for 30 s and 72°C for 30 s; 72°C for 5 min. Reactions were performed in a 2720 Applied BioSystems PCR Thermal Cycler (Life Technologies, USA). The PCR products were verified to electrophoresis analysis in 2% agarose gel.

### Las TaqMan probe-iiPCR assay

The primer pair (Las-F: 5’-AGGTTGGCTGTGTTAAATTTTTTTAAGCAA-3’ and Las-R: 5’-ACAATAACCGAAACCAAAACCTCACT-3’) and TaqMan probe (Las-probe: 5’-6-FAM-ACGGCCAGAATATCTT-MGB-NFQ-3’) were used for Las TaqMan probe-iiPCR assay. These primers/TaqMan probe targeting the Las *sec*E gene region of the *rm*U*-tuf*B-*sec*E*-nus*G*-rpl*KAJL*-rpo*B gene cluster region were confirmed to be Las-specific by Feng et al. [27]. A total volume of 50 μL iiPCR reaction mixture containing 1X Uni-ii HS Buffer (GeneReach), 500 nM of Las-F primer, 500 nM of Las-R primer, 200 nM of Las-probe (ABI, Life Technologies), 5 U of iiPCR Taq DNA Polymerase (GeneReach) and 2 μL template DNA were transferred to the R-tube (GeneReach). The optimized probe concentration was evaluated from 0 nM to 250 nM (0 nM, 50 nM, 100 nM, 150 nM, 200 nM and 250 nM). The iiPCR was performed in the POCKIT Micro Duo Nucleic Acid Analyzer (GeneReach) with the default thermal conditions. The fluorescence signal-to-noise (S/N) ratios (signal_after_/signal_before_) were collected and measured by the POCKIT Micro Duo Nucleic Acid Analyzer. The results according to the default S/N threshold were shown automatically as “+” symbol (S/N ≥ 1.3) or “-” symbol (S/N < 1.3) on the display screen at the end of the program.

### Analytical specificity test of Las TaqMan probe-iiPCR assay

To evaluate the analytical specificity of the Las TaqMan probe-iiPCR assay, the DNA samples from the HLB-affected citrus cultivars (including PM, LSO, EL and WP) were used, and DNAs from healthy plants were included as negative controls. The DNA of common citrus pathogens in Taiwan including *Diaporthe citri* (melanose), *Elsinoe fawcettii* (scab), *Guignardia citricarpa* (black spot), *Mycospharella citri* (greasy spot) and *Xanthomonas citri* (citrus canker) were also evaluated.

### Analytical sensitivity test of Las TaqMan probe-iiPCR assay

To determine the 100% detection end point of the Las TaqMan probe-iiPCR assay, 10-folds serial dilutions of the plasmids containing the Las-primers/probe targeting region (10^4^, 10^3^, 10^2^, 10^1^, 10^0^ copies per reaction) and extracted DNAs from HLB-affected citrus cultivar PM (200 ng, 20 ng, 2 ng, 200 pg, 20 pg and 2 pg per reaction) were subjected to comparative evaluation between the iiPCR assay and a previously described real-time PCR method [27]. All the iiPCR reactions were performed in six replicates with water (no template) controls. The plasmid was constructed with the Las-primer pair amplified PCR product and pCR2.1-TOPO vector, and then transformed into DH5α competent cells according to the manufacturer’s instructions (Invitrogen, USA).

### Detection of Las in field citrus samples

**Fig 1:**
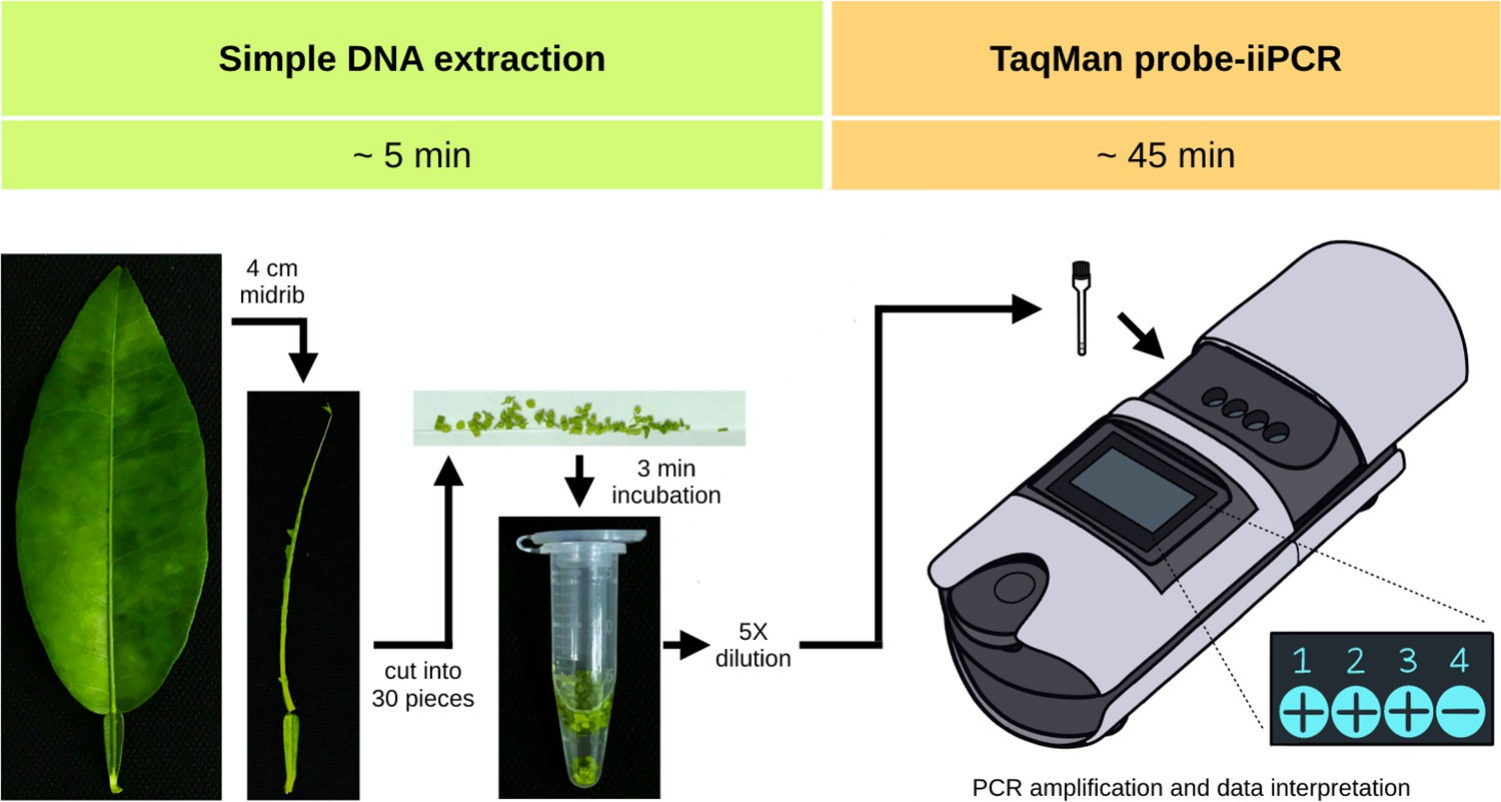
Schematic of the on-site detection system for *Candidatus* Liberibacter asiaticus (Las) detection.

To validate the feasibility of on-site detection system (simple DNA extraction coupled with Las TaqMan probe-iiPCR assay) (Fig 1), the detection of Las in field citrus samples was conducted with citrus trees with and without HLB-like symptoms. A total of 70 samples were collected from citrus cultivars including PM, LSO, EL, and WP in different citrus-growing areas across Taiwan. These samples were also subjected to the conventional DNA extraction coupled with Las TaqMan probe-iiPCR and real-time PCR for comparison. The 2 × 2 table were used for comparing the diagnostic performance of the on-site detection system and TaqMan probe-iiPCR assay. The diagnostic sensitivity, diagnostic specificity, positive predictive value (PPV) and negative predictive value (NPV) were calculated using MedCalc (MedCalc Software Ltd, Belgium).

## Results and discussion

### Optimization of the Las TaqMan probe-iiPCR assay

**Fig 2:**
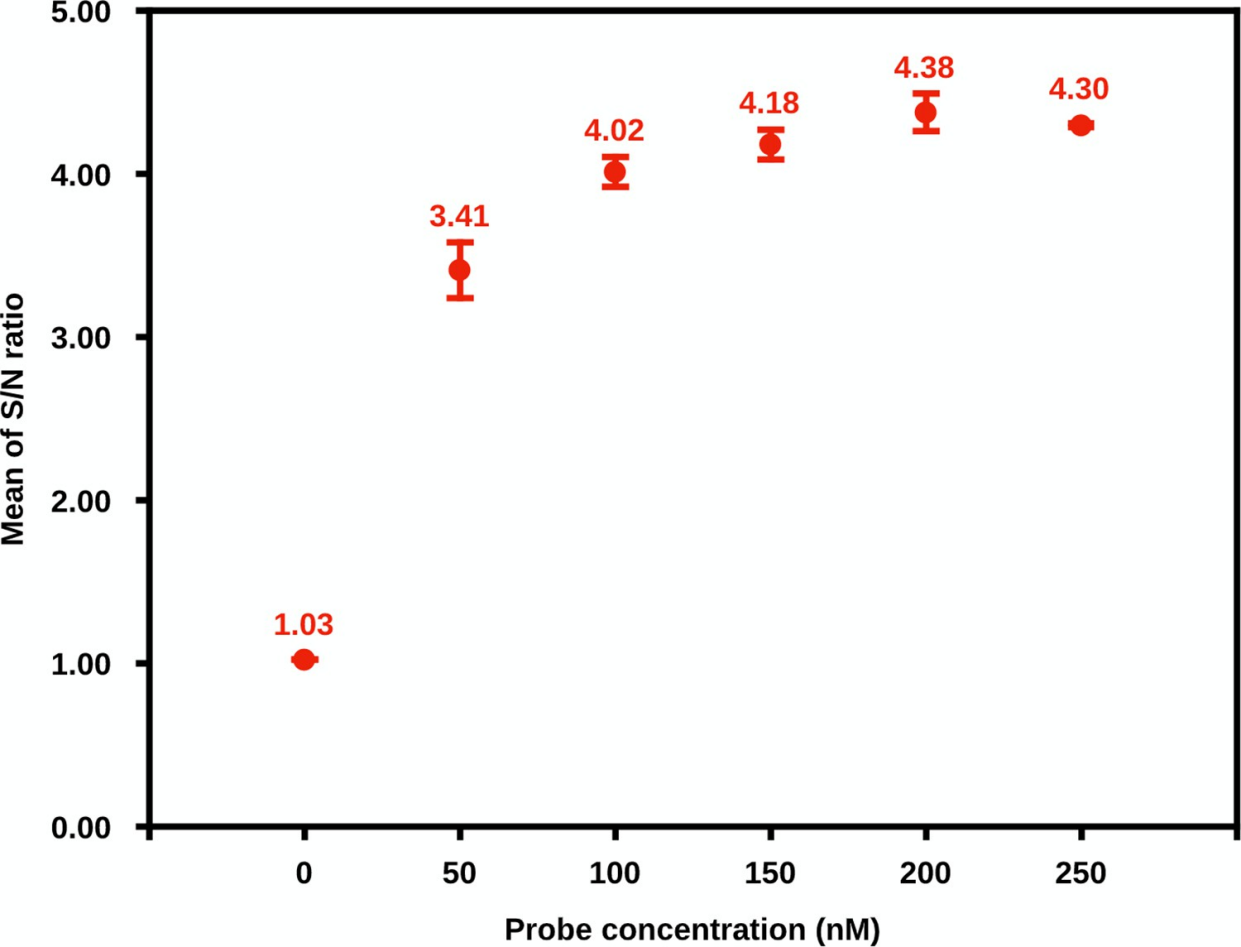
Optimization of concentration of TaqMan probe for iiPCR assay for *Candidatus* Liberibacter asiaticus (Las). Different probe concentrations (0 nM to 250 nM) were evaluated the effects of TaqMan probe concentration on production of fluorescent signals. Mean S/N ratio of each reaction was plotted against probe concentration. Each reaction was performed in three replicate reactions. Error bars represent the SDs. S/N ratio, fluorescent signal_after_/fluorescent signal_before_; SD, standard deviation.

To optimize the target amplification and signal production of Las TaqMan probe-iiPCR assay, different probe concentrations (0 nM to 250 nM) were tested. As shown in Fig 2, S/N ratios increased with TaqMan probe concentrations from 50 nM to 200 nM. The highest S/N ratio of 4.38 indicated that the optimized concentration of Las-probe was 200 nM for the iiPCR assay.

### Analytical specificity evaluation of the Las TaqMan probe-iiPCR assay

The extracted DNA from Las-infected plants, healthy plants and five citrus pathogens (*Diaportha citri*, *Elsinoe fawcettii*, *Guignardia citricarpa*, *Mycospharella citri* and *Xanthomonas citri*) were tested for analytical specificity of Las TaqMan probe-iiPCR assay. All the Las-infected samples were showed positive with mean S/N ratios from 3.11 to 4.13, while the healthy samples showed negative results. The Las TaqMan probe-iiPCR assay was negative for the non-Las pathogens (Table 1). In addition, the Las TaqMan probe-iiPCR assay offered high specificity to detect Las in the HLB-affected citrus plants (Table 1), and the Las TaqMan probe-iiPCR assay did not detect Las in healthy citrus plants kept in the greenhouse also supporting the specificity of the Las TaqMan probe-based iiPCR [28]. Because Las is the only *Ca*. Liberibacter bacterium present in Taiwan, the specificity test of this iiPCR method on other *Ca*. Liberibacter bacteria (Laf and Lam) can only be evaluated *in silico*. The alignments of targeting gene region among three HLB pathogens (Las, Laf and Lam) showed these primer pair (Las-F/Las-R) and TaqMan probe (Las-probe) would be specific to the Las (S1 Fig). Therefore, the success of iiPCR on detecting Las may exemplify the potentials for detecting other *Ca*. Liberibacter species (Laf and Lam) in different citrus-growing regions.

**Table 1:** Analytical specificity analysis of TaqMan probe-iiPCR for *Candidatus* Liberibacter asiaticus (Las) detection.

Tested sample		
Citrus plant^a, b^		Mean of S/N ratio (± SD) ^c^
PM	Las-infected	4.09 ± 0.29
	Healthy	1.07 ± 0.06
LSO	Las-infected	4.13 ± 0.34
	Healthy	1.02 ± 0.03
EL	Las-infected	3.11 ± 0.36
	Healthy	1.06 ± 0.04
WP	Las-infected	3.54 ± 0.43
	Healthy	1.05 ± 0.07
**Non-Las pathogen**	*Diaporthe citri*	1.11 ± 0.06
	*Elsinoe fawcettii*	1.03 ± 0.04
	*Guignardia citricarpa*	1.07 ± 0.04
	*Mycospharella citri*	1.07 ± 0.09
	*Xanthomonas citri*	1.04 ± 0.04

^a^The citrus cultivars included Ponkan mandarin (PM), Liucheng sweet orange (LSO), Eureka lemon (EL) and Wentan pummelo (WP).

^b^Las-infection was confirmed by PCR assay as described in materials and methods.

^c^All the averages of S/N ratio were determined from each three tested samples.

### Analytical sensitivity evaluation of the Las TaqMan probe-iiPCR assay

Serial dilutions of plasmids with the Las-targeting region (10^4^ to 10^0^ copies per reaction) and DNA templates from the Las-infected citrus cultivar PM (200 ng to 2 pg per reaction) were used to evaluate analytical sensitivity of the Las TaqMan probe-iiPCR assay by comparison with the real-time PCR method. As shown in Table 2, the limit of detection for the plasmid was at 10^0^ copy/reaction with the average S/N ratio of 1.50 ± 0.05 by the Las TaqMan probe-iiPCR assay. The Las TaqMan probe-iiPCR assay showed detectable lowest concentration of extracted DNA at 10^−3^ dilution (200 pg), which containing Las amount at 1.7 × 10^0^ copy/reaction based on the real-time PCR quantification (S2 Fig), with the average S/N ratio of 1.60 ± 0.27 and no positive signals were detected with the subsequent diluted DNA (Table 2). Therefore, the 100% detection end points were found at around 1 copy of Las per reaction with both TaqMan probe-iiPCR assay and real-time PCR method. The data indicated that the Las TaqMan probe-iiPCR assay could detect its target with sensitivity comparable to that of the real-time PCR method. The published TaqMan-iiPCR methods for other pathogens detection, such as *Fusarium oxysporum* f. sp. *cubense* race 4, Zika virus and dengue virus, were all showed comparable sensitivity to reference real-time PCR methods [23, 24, 29].

**Table 2:** Analytical sensitivities of TaqMan probe-iiPCR and real-time PCR for detection of *Candidatus* Liberibacter asiaticus (Las).

**Tested sample**	**Copies (per reaction)**	**TaqMan probe-iiPCR assay**		**Real-time PCR method**
		**Detection results (no. positive/no. total)**	**Mean of S/N ration (± SD)** ^**a**^	**Ct mean (± SD)** ^**b**^
Plasmid^c^	10^4^	+ + + + + + (6/6)	4.47 ± 0.06	25.28 ± 0.03
	10^3^	+ + + + + + (6/6)	4.35 ± 0.04	28.75 ± 0.02
	10^2^	+ + + + + + (6/6)	3.62 ± 0.05	32.18 ± 0.06
	10^1^	+ + + + + + (6/6)	2.58 ± 0.03	35.80 ± 0.41
	10^0^	+ + + + + + (6/6)	1.50 ± 0.05	39.33 ± 0.18
	NTC	- - - - - - - (0/6)	1.02 ± 0.01	Und
**Tested sample**	**DNA amount (per reaction)**	**TaqMan probe-iiPCR assay**		**Real-time PCR method**
		**Detection results (no. positive/no. total)**	**Mean of S/N ration (± SD)** ^**a**^	**Ct mean (± SD)** ^**b**^
Extracted DNA^d^	200 ng	+ + + + + + (6/6)	4.36 ± 0.08	28.53 ± 0.08
	20 ng	+ + + + + + (6/6)	3.77 ± 0.22	31.86 ± 0.11
	2 ng	+ + + + + + (6/6)	2.53 ± 0.20	35.20 ± 0.42
	200 pg	+ + + + + + (6/6)	1.60 ± 0.27	38.76 ± 0.57
	20 pg	- - - - - - - (0/6)	1.05 ± 0.07	Und
	2 pg	- - - - - - - (0/6)	1.03 ± 0.02	Und
	NTC	- - - - - - - (0/6)	1.02 ± 0.01	Und

^a^All the means of S/N ratio were determined from each six tested samples.

^b^The average Ct value was determined with triplicate for each sample.

^c^The plasmids contained the Las-primers/probe targeting region.

^d^The DNA was extracted from the Las-infected citrus plant (cultivar Ponkan mandarin).

Boxed, 100% detection end point; NTC, no template control (ddH_2_O); S/N ratio, fluorescent signal_after_/fluorescent signal_before_; SD, standard deviation; Und, undetermined.

### Optimization of the simple DNA extraction procedure

**Fig 3:**
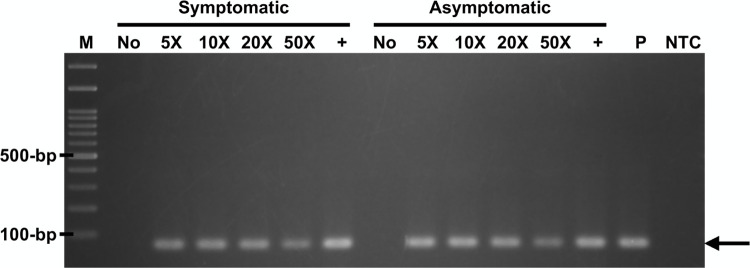
Dilution ratio evaluation of simple DNA extraction by PCR assay. The plant 18S rRNA-encoding gene amplicons (67-bp, arrow) were detected in electrophoresis analysis. M, 100-bp DNA ladders (GeneDireX); No, no dilution; 5X, 5-folds dilution; 10X, 10-folds dilution; 20X, 20-folds dilution; 50X, 50-folds dilution; +, DNA isolated by conventional extraction; P, 10^3^ copies of standard template (plasmid containing targeting gene region); NTC, no template control (ddH_2_O).

Different dilution (5-folds to 50-folds) of the sap in TE buffer were evaluated by PCR assay for the extraction method optimization. The expected amplicons of plant 18S rRNA-encoding gene were observed from both symptomatic and asymptomatic samples. Among all the diluted samples, 5-folds dilution showed slightly better results (Fig 3). Therefore, 5-folds dilution was used in following assay.

### Field detection by the on-site Las detection system

To evaluate whether the on-site detection system (simple DNA extraction coupled with Las TaqMan probe-iiPCR assay) (Fig 1) was suitable for the detection of Las in fields, a total of 70 citrus samples (including 54 HLB-like symptoms samples and 16 healthy-looking samples) from four main cultivars (PM, LSO, EL and WP) were collected. The DNA (isolated by conventional extraction) of these field samples were also tested using the Las TaqMan probe-iiPCR assay and real-time PCR method for comparison. Among all 70 samples, the Las detection rates were 71.4% (50/70), 75.7% (53/70) and 75.7% (53/70) by on-site detection system, Las TaqMan probe-iiPCR assay and real-time PCR method, respectively (Table 3). Three samples (one HLB-like symptoms samples and two healthy-looking samples) were Las-positive by Las TaqMan probe-iiPCR assay and real-time PCR method but negative by the on-site detection system (Table 3). As shown in Table 4, the diagnostic sensitivity of the on-site detection system compared with reference real-time PCR method was 94.34% (95% CI 84.34%–98.82%) and the diagnostic specificity of 100% (95% CI 80.49%–100%). The positive predictive value (PPV) of the on-site detection system was 100% and the negative predictive value (NPV) was 85% (95% CI 65.37%–94.45%). There was complete agreement between Las TaqMan probe-iiPCR assay and real-time PCR method, giving a diagnostic sensitivity of 100% (95% CI 93.28%–100%), diagnostic specificity of 100% (95% CI 80.49%–100%), PPV of 100% and NPV of 100%. The results demonstrated that both the on-site detection system and TaqMan probe-iiPCR assay had great performances in Las detection. Three healthy-looking citrus samples was Las-positive by Las TaqMan probe-iiPCR assay and real-time PCR method. These citrus samples might be at the early stage of HLB infection. The latent period of HLB is long and infected citrus plants can be asymptomatic from months to years [30, 31]. Previous studies showed that the asymptomatic leaves of HLB-affected plants have low Las titer [32, 33]. Therefore, it is important to enable early detection of Las as the diseased citrus trees during the latent period are potential sources to spread the disease. The field evaluation of the on-site detection system showed a highly sensitivity, which would be useful to identify infected plants in early infection. Worth noticing, there were citrus samples detected Las-positive by the TaqMan probe-iiPCR assay in the lab, but negative by the on-site detection system in fields. The different results between might due to the efficiency of simple DNA extraction and low amounts of Las in the on-site field samples. According to the real-time PCR quantification results, these ambiguous field samples generally had Las titer around 10^1^ copies. Therefore, the final subjected DNA amount might be lower than the detection limit of the iiPCR assay. In summary, the developed on-site detection system and Las TaqMan probe-iiPCR assay can be alternative methods for Las detection with great reliability.

**Table 3:** Detection of *Candidatus* Liberibacter asiaticus (Las) in field citrus samples by on-site detection system, TaqMan probe-iiPCR assay and real-time PCR method.

		On-site detection system^a^		TaqMan probe-iiPCR assay^b^		Real-time PCR method^c^	
Tested samples^d^	No.	Positive	Negative	Positive	Negative	Positive	Negative
Symptomatic	54	49	5	50	4	50	4
Asymptomatic	16	1	15	3	13	3	13
Total	70	50	20	53	17	53	17

^a^The on-site detection system was performed as simple DNA extraction with Las-probe iiPCR.

^b^The TaqMan probe-iiPCR assay was performed as conventional DNA extraction with Las-probe iiPCR.

^c^The real-time PCR method was carried out as described in Feng et al [27].

^d^Samples were collected from citrus trees with HLB-like symptoms (symptomatic) varying from mild chlorotic to typical blotchy mottling or from healthy-looking (asymptomatic) citrus trees.

**Table 4:** Comparison of diagnostic performances of on-site detection system, TaqMan probe-iiPCR assay with real-time PCR method for *Candidatus* Liberibacter asiaticus (Las) detection.

		**Real-time PCR method** ^**a**^		
		Positive	Negative	
**On-site detection system** ^**b**^	Positive	50	0	50
	Negative	3	17	20
		53	17	
**Sensitivity**	**Specificity**	**PPV**	**NPV**	
94.34% (95% CI 84.34%–98.82%)	100% (95% CI 80.49%–100%)	100%	85% (95% CI 65.37%–94.45%)	
		**Real-time PCR method** ^**a**^		
		Positive	Negative	
**TaqMan probe-iiPCR assay** ^**c**^	Positive	53	0	53
	Negative	0	17	17
		53	17	
**Sensitivity**	**Specificity**	**PPV**	**NPV**	
100% (95% CI 93.28%–100%)	100% (95% CI 80.49%–100%)	100%	100%	

^a^The real-time PCR method was carried out as described in Feng et al [27].

^b^The on-site detection system was performed as simple DNA extraction with Las-probe iiPCR.

^c^The TaqMan probe-iiPCR assay was performed as conventional DNA extraction with Las-probe iiPCR.

Abbreviation: CI, confidence interval; PPV, positive predictive value; NPV, negative predictive value.

## Conclusion

HLB is one of the most important citrus diseases and has caused significant economic damages to the citrus industry around the world [1]. Because there are no cures for HLB currently, the control of HLB is based on HLB-tolerant rootstock cultivars, healthy seedlings, removing infected plant hosts in the field, and preventing insect vectors. Sensitive detection methods for HLB diagnosis not only play an important role in these control strategies, but also can be useful for understanding the disease ecology to achieve better management of HLB disease [28]. The present study provided a TaqMan probe-based iiPCR assay on a rapid and sensitive field-deployable platform, enabling the detection of Las near the citrus plants to allow a timely response to manage the disease. Therefore, working on the field-deployable POCKIT device, the Las TaqMan probe-based iiPCR assay has potential to serve as a rapid and simple near point-of-need tool for routine detection of Las in citrus samples. In summary, this on-site detection system could shorten the time and labor costs for Las detection and will be useful in improving HLB management.

## Supporting information

S1 Fig The alignment of *Candidatus* Liberibacter asiaticus (Las)-specific primers/probe targeting regions among Las, *Ca*. L. africanus (Laf) and *Ca*. L. americanus (Lam). Reference genomes: Las (CP019958.1, position 25180–25274), Laf (CP004021.1, position 909639–909733) and Lam (CP006604.1, position 518568–518662).
(PDF)

S2 Fig The standard curve of real-time PCR for *Candidatus* Liberibacter asiaticus (Las) absolute quantification. The 10-fold serial dilutions (10^6^−10^1^ copies) of plasmid DNA (with the targeting Las *sec*E fragment) were used for developing the standard curve.
(PDF)

S1 Raw images (PDF)
