# Supplementary material for: On-site detection system of Candidatus Liberibacter asiaticus by using TaqMan probe-based insulated isothermal polymerase chain reaction (iiPCR)
Source: PLoS One. 2023 Jun 23;18(6):e0287699. doi: 10.1371/journal.pone.0287699 (PMC10289410; doi:10.1371/journal.pone.0287699)
Supplement: S1 Fig — Reference genomes: Las (CP019958.1, position 25180–25274), Laf (CP004021.1, position 909639–909733) and Lam (CP006604.1, position 518568–518662). (PDF) [file pone.0287699.s001.pdf]

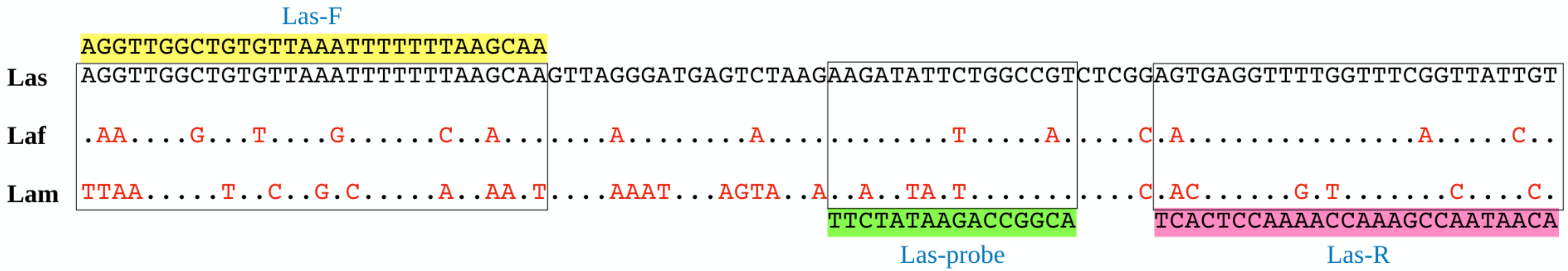

S1 Fig. The alignment of *Candidatus* Liberibacter asiaticus (Las)-specific primers/probe targeting regions among Las, *Ca*. L. africanus (Laf) and *Ca*. L. americanus (Lam). Reference genomes: Las (CP019958.1, position 25180–25274), Laf (CP004021.1, position 909639–909733) and Lam (CP006604.1, position 518568–518662).
