# Supplementary material for: On-site detection system of Candidatus Liberibacter asiaticus by using TaqMan probe-based insulated isothermal polymerase chain reaction (iiPCR)
Source: PLoS One. 2023 Jun 23;18(6):e0287699. doi: 10.1371/journal.pone.0287699 (PMC10289410; doi:10.1371/journal.pone.0287699)
Supplement: S2 Fig — The 10-fold serial dilutions (106−101 copies) of plasmid DNA (with the targeting Las secE fragment) were used for developing the standard curve. (PDF) [file pone.0287699.s002.pdf]

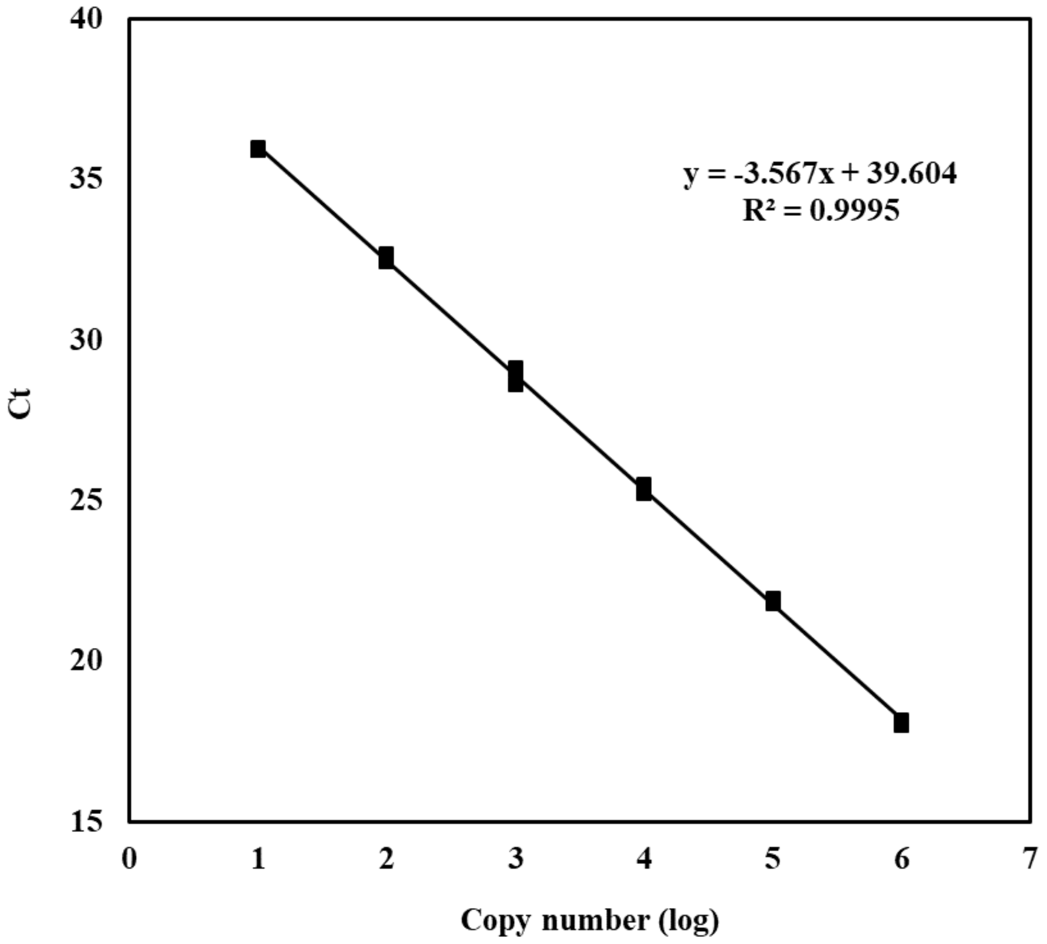

S1 Fig. The standard curve of real-time PCR for *Candidatus* Liberibacter asiaticus (Las) absolute quantification. The 10-fold serial dilutions (10^6-10^1 copies) of plasmid DNA (with the targeting Las *sec*E fragment) were used for developing the standard curve.
