# Supplementary material for: On-site detection system of Candidatus Liberibacter asiaticus by using TaqMan probe-based insulated isothermal polymerase chain reaction (iiPCR)
Source: PLoS One. 2023 Jun 23;18(6):e0287699. doi: 10.1371/journal.pone.0287699 (PMC10289410; doi:10.1371/journal.pone.0287699)
Supplement: S1 Raw images — (PDF) [file pone.0287699.s003.pdf]

M: marker (DNA ladder)
1-14: experimental samples

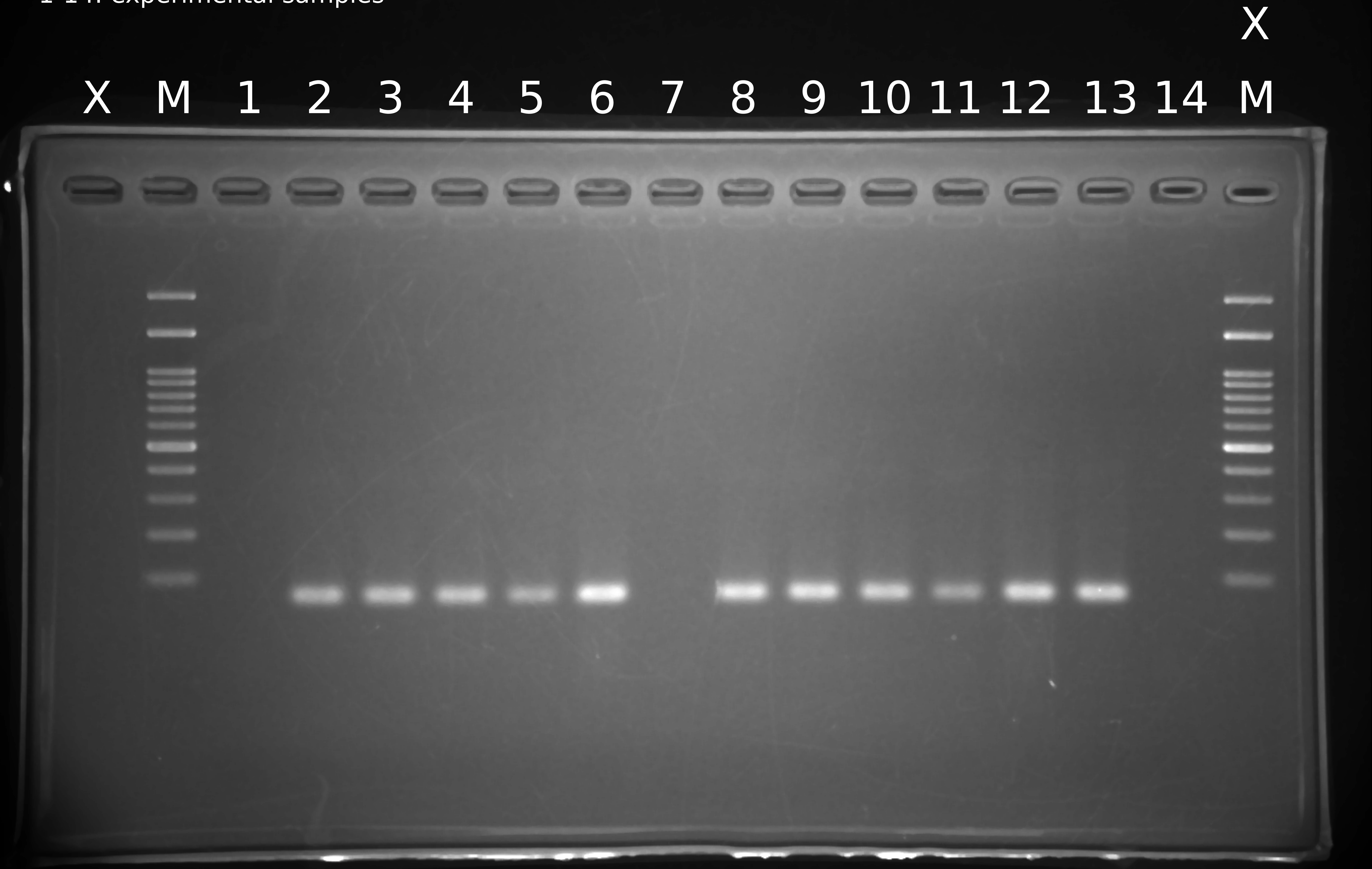

Gel was stained with EtBr and visualized under an UV densitometer supplied by AlphaEaseFC Image Analysis Software.
